# Detection of early-stage Alzheimer’s pathology using blood-based autoantibody biomarkers in elderly hip fracture repair patients

**DOI:** 10.1371/journal.pone.0225178

**Published:** 2019-11-15

**Authors:** Cassandra DeMarshall, Esther Oh, Rahil Kheirkhah, Frederick Sieber, Henrik Zetterberg, Kaj Blennow, Robert G. Nagele

**Affiliations:** 1 Biomarker Discovery Center, New Jersey Institute for Successful Aging, Rowan University School of Osteopathic Medicine, Stratford, New Jersey, United States of America; 2 Department of Geriatrics and Gerontology, Rowan University School of Osteopathic Medicine, Stratford, New Jersey, United States of America; 3 Department of Medicine, Psychiatry and Behavioral Sciences, Pathology, Johns Hopkins University School of Medicine, Baltimore, MD, United States of America; 4 Department of Anesthesiology and Critical Care Medicine, Johns Hopkins Bayview Medical Center, Baltimore, Maryland, United States of America; 5 Graduate School of Biomedical Sciences (GSBS), Rowan University, Stratford, New Jersey, United States of America; 6 Department of Psychiatry and Neurochemistry, Institute of Neuroscience and Physiology, the Sahlgrenska Academy at the University of Gothenburg, Mölndal, Sweden; 7 Clinical Neurochemistry Laboratory, Sahlgrenska University Hospital, Mölndal, Sweden; 8 Department of Neurodegenerative Disease, UCL Queen Square Institute of Neurology, Queen Square, London, England, United Kingdom; 9 UK Dementia Research Institute at UCL, London, England, United Kingdom; Banner Alzheimer's Institute, UNITED STATES

## Abstract

Post-operative delirium (POD) is the most common complication following major surgery in non-demented older (>65 y/o) patients. Patients experiencing POD show increased risk for future cognitive decline, including mild cognitive impairment (MCI) and Alzheimer’s disease (AD) and, conversely, patients with cognitive decline at surgery show increased risk for POD. Here, we demonstrate that a previously established panel of AD-driven MCI (ADMCI) autoantibody (aAB) biomarkers can be used to detect prodromal AD pre-surgically in individuals admitted into the hospital for hip fracture repair (HFR) surgery. Plasma from 39 STRIDE (STRIDE: A Strategy to Reduce the Incidence of Postoperative Delirium in Elderly Patients) HFR patients and sera from 25 age- and sex-matched non-demented and non-surgical controls were screened using human protein microarrays to measure expression of a panel of 44 previously identified MCI aAB biomarkers. The predictive classification accuracy of the aAB biomarker panel was evaluated using Random Forest (*RF*). The ADMCI aAB biomarkers successfully distinguished 21 STRIDE HFR patients (CDR = 0.5) from 25 matched non-surgical controls with an overall accuracy of 91.3% (sensitivity = 95.2%; specificity = 88.0%). The ADMCI aAB panel also correctly identified six patients with preoperative CDR = 0 who later converted to CDR = 0.5 or >1 at one-year follow-up. Lastly, the majority of cognitively normal (CDR = 0) STRIDE HFR subjects that were positive for CSF AD biomarkers based on the A/T/N classification system were likewise classified as ADMCI aAB-positive using the biomarker panel. Results suggest that pre-surgical detection of ADMCI aAB biomarkers can readily identify HFR patients with likely early-stage AD pathology using pre-surgery blood samples, opening up the potential for early, blood-based AD detection and improvements in peri- and postoperative patient management.

## Introduction

AD is a progressive neurodegenerative disease afflicting approximately 5.8 million people in the US, including almost half of those at 85 years and older [1–3]. A clear understanding of AD pathogenesis and the factors and/or conditions that trigger it remains elusive and controversial. Available treatments can only temporarily alleviate some symptoms but do not reverse or slow pathological progression [4]. It is now generally agreed that AD-related neuropathological changes can begin in the brain as early as 20 years before symptoms appear and can be evaluated for diagnosis and potential treatment [5–11]. This delay in emergence of symptoms after the onset of pathology makes it difficult to identify AD patients at earlier, preclinical (or pre-symptomatic) and prodromal (mild cognitive impairment, MCI) disease stages, i.e., at times when disease-modifying treatments would likely be most effective and beneficial.

Currently, diagnosis of AD is mostly based on a combination of medical history, detailed physical, neurological and neuropsychological examinations and sometimes brain imaging, but earlier preclinical and prodromal stages are difficult to diagnose accurately using these methods [8, 11]. In recent years, a number of soluble components in blood and cerebrospinal fluid (CSF) have been identified that have potential as useful biomarkers for AD [12]. In CSF, amyloid-beta_1-42_ (Aβ42), total tau (t-tau) and phosphorylated tau (p-tau) and their relative ratios have been linked to different aspects of AD pathology [13–18]. For example, low CSF Aβ42 levels in individuals with mild cognitive impairment (MCI) correlate well with brain amyloidosis as detected by Aβ PET imaging and is now considered to be a strong and reliable indicator of the presence of early ongoing AD pathology in individuals with prodromal AD (MCI) as well as a good predictor of rapid progression to full-blown AD [19–24]. Likewise, CSF levels of phosphorylated tau (p-tau) and total tau (t-tau), biomarkers of tau pathology and neurodegeneration or injury, respectively, have been used successfully together or in combination with Aβ42 as positive indicators of existing AD pathology [18, 25].

Despite their high overall accuracy and direct linkage to AD pathology, a practical limitation of using CSF is the requirement for lumbar puncture, which is considered somewhat invasive and not without risk [16, 21, 22]. Because procurement of blood is much less invasive and associated with less risk, intense research efforts are currently underway worldwide to identify blood-based biomarkers, directly linked to AD pathology, aiming for an overall accuracy at least comparable to the more established CSF biomarkers [26]. Plasma proteins [27–29], lipids [30], Aβ42 and tau [31, 32] as well as proteins and microRNAs enclosed within exosomes and lysosomal derivatives have all been showing promise as biomarkers for early detection of AD pathology [33, 34]

Our previous studies using human protein microarrays have shown that aABs are abundant and ubiquitous in the blood, and that individual autoantibody profiles are influenced by age, gender and the presence or absence of disease [35]. We have also identified common changes in aAB profiles that appear to have utility as biomarkers for several neurodegenerative diseases, including AD, Parkinson’s disease (PD) and multiple sclerosis [36–40]. In a recent proof-of-concept study, serum samples obtained from subjects enrolled in the Alzheimer’s Disease Neuroimaging Initiative (ADNI) were analyzed using protein microarrays in an effort to identify aAB biomarkers associated with MCI due to underlying AD etiology. These MCI subjects were clinically well-characterized, including documentation of CSF Aβ42, p-tau and t-tau levels consistent with the likely presence of existing, early-stage AD pathology. A panel of selected ADMCI aAB biomarkers derived from analysis of serum from these ADNI subjects was found to be capable of distinguishing ADNI MCI subjects from cognitively normal individuals as well as from patients with more advanced, mild-moderate AD and/or other neurodegenerative and non-neurodegenerative diseases with high overall accuracy [37].

To further explore the utility of the panel of the ADMCI aAB biomarkers derived from the analysis of ADNI subjects, we tested its accuracy using another cohort of patients participating in a randomized controlled trial (STRIDE: A Strategy to Reduce the Incidence of Postoperative Delirium in Elderly Patients, ClinicalTrials.gov: NCT00590707) involving hip fracture repair (HFR). Previous studies on STRIDE HFR subjects have supported an association between AD pathology, gait disorders and frequent falls leading to hip fracture [41, 42]. This work has also shown that a large proportion of elderly HFR patients without dementia have abnormal CSF Aβ42, t-tau and p-tau levels, indicating the likely presence of early-stage AD pathology [42]. In the present study, our goals were to determine (1) the utility of a previously established panel of blood-based ADMCI aAB biomarkers, derived from well-characterized ADNI subjects with amnestic MCI, for distinguishing STRIDE participants admitted into the hospital for HFR surgery with cognitive impairment (CDR = 0.5) from age-and sex-matched non-demented controls, and (2) if there is a consistent relationship between measured levels of CSF biomarkers indicative of existing AD pathology and ADMCI aAB biomarkers in the blood of the same subjects.

## Materials and methods

This study was approved by the Rowan University Institutional Review Board (IRB) and the Johns Hopkins University IRB which oversaw the STRIDE study.

### Blood collection and processing

*S*erum and plasma samples studied were from a total of 64 subjects, STRIDE participants and non-demented controls obtained from *Bioserve Biotechnologies*, *Ltd* as described in detail below. All samples were collected using standard procedures and stored at −80°C until use. Stored samples were monitored using a Sensaphone 1400 (Phonetics, Inc., Aston, PA).

### CSF collection and processing

CSF samples were collected at the procedure for routine spinal anesthesia, before the injection of the anesthetics, aliquoted and stored at −80°C.

### STRIDE participants (n = 39) (Table 1)

Hip fracture patients enrolled in the randomized clinical trial “A Strategy to Reduce the Incidence of Postoperative Delirium in Elderly Patients” (STRIDE) [41] were analyzed. A detailed description of the STRIDE study has been published previously [41, 43]. Briefly, inclusion criteria were age ≥65, preoperative MMSE score ≥15, and eligible for spinal anesthesia. Main exclusion criteria were preoperative delirium, stage IV congestive heart failure, or severe chronic obstructive pulmonary disease. Informed consent was obtained from patients or appropriate legal representatives for patients unable to give informed consent due to cognitive impairment. STRIDE HFR subjects were cognitively assessed [41] prior to surgery using both a mini-mental status exam (MMSE) [44] and a modified CDR as described previously [42]. Blood was taken pre-surgically and CSF was obtained during administration of spinal anesthesia, which allowed measurement and evaluation of key CSF AD biomarkers levels.

**Table 1:** Baseline demographics.

	STRIDE Subjects Controls			
Clinical Dementia Rating (CDR)				
	Total	0	0.5	n/a
	(n = 39)	(n = 18)	(n = 21)	(n = 25)
**Demographics**				
**Age, mean (SD)**	80.9(8.1)	77.8(7.1)	83.5(8.1)	67.7(6.2)
**Sex, male, n(%)**	8(21)	4(22)	4(19)	12(48)
**female, n(%)**	31(79)	14(78)	17(81)	13(52)

**Table 1.** The number of individuals (n), age, gender, and ethnicity are listed for each group. For all HFR and MCI subjects, the Clinical Dementia Rating (CDR) score is included as a measure of cognitive impairment. CDR score is not available for Bioserve control subjects.

Demographic data were collected from patients, informants, and medical records. Prior to surgery, trained research staff obtained history from patients and their informants. The research staff also administered the MMSE to patients and the Short Form of the Informant Questionnaire on Cognitive Decline in the Elderly (Short IQCODE) to the family or caregivers [45] for the STRIDE patients. A consensus panel of two psychiatrists and one geriatrician, blinded to the intervention, scored the CDR. CDR scoring was based on assessment of all available clinical and cognitive data, as well as the Short IQCODE [45] and other history collected from the patient and informant prior to surgery.

### Non-demented, non-surgical controls (n = 25) (Table 1)

Control sera were obtained from *Bioserve Biotechnologies Ltd*. The samples were from healthy patients, age- and sex-matched as closely as possible to the STRIDE cohort.

### CSF Biomarkers

CSF samples were analyzed for Aβ40, Aβ42, phosphorylated tau (p-tau) and total tau (t-tau). Classification cutoffs used here to define abnormal biomarker levels associated with underlying, ongoing AD pathology in the STRIDE cohort have been described previously [42]. Briefly, the optimal cutoff ratio of CSF Aβ42/Aβ40 which was closely correlated with abnormal Aβ PET was determined previously to be ≤ 0.8 (CSF Aβ42/40 ratio x 10) [19]. CSF p-tau, the biomarker of tau pathology, was classified as normal if the level was ≤ 60 pg/ml or abnormal if ≥ 60 pg/ml. The CSF biomarker of neurodegeneration and neuronal injury, t-tau, was categorized as normal if levels were ≤ 350 pg/ml or abnormal if ≥ 350 pg/ml. The relationships between CSF biomarker levels, CDR scores and a designation of MCI+ or MCI- based on ADMCI aAB biomarkers was evaluated.

In individuals without dementia (CDR = 0 or 0.5), CSF biomarkers were further divided into categories according to the A/T/N classification system, where “A” refers to the value of an Aβ biomarker,”T” the value of a tau biomarker, and “N” the value of a neurodegeneration biomarker [46]. Using this system, the CSF Aβ42/40 ratio was classified as normal (A-) or abnormal (A+), p-tau as normal (T-) or abnormal (T+), and t-tau as normal (N-) or abnormal (N+) based on the cutoff values mentioned above. A/T/N classifications were also coupled to the National Institute on Aging-Alzheimer’s Association (NIA-AA) criteria as described previously [47].

### Human protein microarrays for detection of autoantibodies (aABs)

Invitrogen's ProtoArray v5.1 Human Protein Microarrays (Cat. No. PAH0525020, Invitrogen, Carlsbad, CA, USA), each containing 9486 unique human protein antigens (www.invitrogen.com/protoarray), were used for aAB detection. All proteins were expressed as glutathione s-transferase (GST) fusion proteins in insect cells, purified under native conditions, and spotted in duplicate onto nitrocellulose-coated glass slides. Arrays were probed with serum/plasma, processed and scanned according to the manufacturer's instructions. Briefly, microarrays were blocked using Blocking Buffer (Cat. No. PA055, Invitrogen) and each was incubated with serum/plasma diluted to 1:500 in washing buffer. After washing, arrays were probed with anti-human IgG (H + L) conjugated to AlexaFluor 647 (Cat. No. A-21445, Invitrogen) diluted 1:2000 in washing buffer. Arrays were then washed, dried, and immediately scanned with a GenePix 4000B Fluorescence Scanner (Molecular Devices, Sunnyvale, CA, USA).

Fluorescence data were acquired by aligning the Genepix Array List onto the microarray using the Genepix Pro analysis software. The resulting Genepix results files were imported into Invitrogen's *Prospector* 5.2.3 microarray analysis software for analysis. The “group characterization” and “two-group comparison” features in the Immune Response Biomarker Profiling (IRBP) toolbox within *Prospector* enabled M-statistical analysis of differential aAB expression between comparison groups. *Prospector* was used to compare blood autoantibody profiles in the STRIDE and Bioserve cohorts. All data are MIAME compliant and raw data from the microarrays have been deposited in a MIAME compliant database (GEO) under accession number GSE137422.

Subjects were organized into Testing Sets, each consisting of 25 BioServe non-demented controls (matched as closely as possible by age and sex) and one of the following groups: 21 *STRIDE* subjects with CDR = 0.5; 18 STRIDE participants with CDR = 0; and six STRIDE individuals with no cognitive impairment (CDR = 0) at surgery, but who later converted to CDR = 0.5 or CDR≥1 during a one-year follow-up. To evaluate the diagnostic accuracy of the ADMCI aAB biomarker panel described in our previous study [37] (**S1 Table**), the predictive classification accuracy of the selected biomarkers was tested with R 's Random Forest (RF; v 4.6–14) using the default settings [48, 49]. Classification accuracy is reported in a confusion matrix.

## Results

### ADMCI aAB biomarkers in presurgical blood can accurately identify HFR patients with cognitive impairment (CDR = 0.5)

The majority of STRIDE HFR patients were classified either as cognitively normal (CDR = 0) or as mild cognitive impairment (MCI) (CDR = 0.5) (**Table 1**). The expression of 44 previously identified MCI aAB biomarkers (see **S1 Table**) in the blood was determined in CDR = 0.5 HFR subjects and compared with that in non-demented, non-surgical controls using human protein microarrays. Random Forest (*RF*) analysis of the resulting aAB profiles demonstrated that 20 of 21 CDR = 0.5 HFR subjects and 22 of 25 non-demented controls were correctly classified as MCI and controls, respectively (**Table 2**), which translates into an overall accuracy of 91.3% (sensitivity = 95.2%; specificity = 88.0%) (**Table 3**).

**Table 2:** Detection of Mild Cognitive Impairment in STRIDE HFR patients (CDR = 0.5) using AD-driven MCI biomarkers and *Random Forest (RF)* analysis.

	NDC	STRIDE CDR = 0.5
**NDC**	22	3
**STRIDE CDR = 0.5**	1	20

**Table 2**. 21 STRIDE CDR = 0.5 were compared to 25 non-demented controls. Using 44 ADMCI aAB biomarkers, 20/21 patients were identified as MCI, while 22/25 non-demented controls were correctly classified as controls.

**Table 3:** Diagnostic results using a panel of 44 AD-driven MCI biomarkers and Random Forest (*RF*).

Random Forest Analysis	
**Sensitivity %**	95.2
**Specificity %**	88.0
**Positive Predictive Value (PPV) %**	86.7
**Negative Predictive Value (NPV) %**	95.7
**Overall Accuracy %**	91.3
**Overall Error %**	8.7

**Table 3.** Diagnostic performance was assessed using Random Forest (*RF)*. *RF* successfully distinguished STRIDE CDR = 0.5 (n = 21) from non-demented controls (n = 25) with high overall accuracy.

### ADMCI aAB biomarkers can identify cognitively normal (CDR = 0) HFR patients who converted to CDR = 0.5 or CDR>1 at one year post-surgery

From the population of cognitively normal (CDR = 0) hip fracture repair subjects (n = 18), six were found to have converted to either MCI (CDR = 0.5) (n = 5) or AD (CDR≥1) (n = 1) at one year following surgery. In view of this, we next asked if the panel of ADMCI aAB biomarkers could be used to identify cognitively normal individuals who are at high risk for progressing to MCI or dementia at one year follow-up based on aAB profiles obtained from their pre-surgical blood samples. At the time of sampling, only one of the six CDR = 0 patients that converted to MCI or AD in the following year had a CSF A+/T+/N+ profile consistent with a high probability of progression. Plasma samples from these six cognitively normal subjects were compared to plasma from the 21 STRIDE MCI (CDR = 0.5) patients mentioned above using human protein microarrays. Using the same 44 ADMCI aAB biomarkers, all six cognitively normal patients that demonstrated cognitive decline at 1 year follow-up had biomarker profiles that were consistent with MCI (**Table 4**). These results suggest that, although these six patients were initially classified at the time of presurgical blood draw as cognitively normal using CDR, they possessed ADMCI aAB biomarker levels in their blood consistent with the presence of AD-related pathology and a designation of MCI at the time of surgery.

**Table 4:** Presurgical AD detection using AD-driven MCI biomarkers and *Random Forest (RF)* analysis.

	MCI (STRIDE CDR = 0.5)	Converter (STRIDE CDR = 0)
**MCI** **(STRIDE CDR = 0.5)**	21	6
**Converter** **(STRIDE CDR = 0)**	0	0

**Table 4.** Six cognitively normal (CDR = 0) patients that converted to either MCI or AD were compared to the 21 STRIDE CDR = 0.5. Using the same 44 ADMCI aAB biomarkers, all six patients that converted were identified pre-surgically as MCI, suggesting that they had ADMCI aAB biomarker profiles in their blood at levels consistent with the presence of AD-related pathology at the time of surgery.

### ADMCI aAB biomarkers are capable of identifying preclinical AD pathology in otherwise cognitively normal (CDR = 0) STRIDE patients

Our next objective was to determine whether ADMCI aAB biomarkers can be used to identify those with likely underlying AD pathology among the cognitively normal (CDR = 0; preclinical) hip fracture repair subjects (n = 18). To achieve this, we first evaluated CSF biomarker profiles of cognitively normal HFR patients with CDR = 0 according to the A/T/N and corresponding NIA-AA classification system (**Table 5**). Among HFR individuals in the CDR = 0 group, the majority (77.7%, 14/18) had abnormal CSF biomarker profiles consistent with the presence of preclinical AD, with two subjects in the Suspected Non-Alzheimer’s Pathology (SNAP) category and the remaining two with normal CSF biomarker levels. When the panel of 44 ADMCI aAB biomarkers and human protein microarrays were used to compare plasma samples from the same 18 cognitively normal (CDR = 0) individuals with those from 25 non-surgical controls, analysis using *RF* identified 17 of 18 (94.4%) STRIDE CDR = 0 subjects as MCI, with 24 of the 25 (96.0%) controls properly identified as controls (**Table 6**). These results suggest that aABs may be useful as blood-based biomarkers to detect preclinical AD pathology in apparently cognitively normal individuals. Furthermore, in the context of our cohort of HFR subjects, both CSF biomarkers and blood-based ADMCI aAB biomarkers are more sensitive than CDR assessment when applied to detection of early stages of AD pathology.

**Table 5:** CSF biomarker profiles of HFR patients with CDR = 0.5 and CDR 0 using the A/T/N classification system.

CDR 0.5	MCI	MCI		MCI-SNAP
**n = 21**				
** **	(unlikely due to AD)	(A+/T-/N-; A+/T+/N-; A+/T-/N+;		(A-/T+/N-; A-/T-/N+;
** **	(A-/T-/N-)	A+/T+/N+)		A-/T+/N+)
		**% (n/subgroup total)**		
	0	95.2(20/21)		4.7(1/21)
**CDR 0**	Normal	Preclinical AD		SNAP
**n = 18**		**Stage 1**	**Stage 2/3**	
** **	(A-/T-/N-)	(A+/T-/N-)	(A+/T+/N-;	(A-/T+/N-;
** **			A+/T-/N+	A-/T-/N+;
** **			A+/T+/N+)	A-/T+/N+)
		**% (n/subgroup total)**		
	11.1(2/18)	22.2(4/18)	55.5(10/18)	11.1(2/18)

**Table 5.** CSF biomarker profiles of HFR patients without dementia (CDR = 0 and 0.5) were categorized according to the A/T/N and corresponding NIA-AA classification system. The vast majority of patients had CSF biomarkers suggestive of preclinical AD. In the CDR = 0.5 group, 95.2% (20/21) of patients had abnormal CSF biomarker levels. The remaining patient was categorized as Suspected Non-Alzheimer’s Pathology (SNAP). In the CDR = 0 group, 11.1% (2/18) samples had normal biomarker levels, while 77.7% (14/18) of patients had abnormal biomarker levels. Cutoff values for each biomarker level considered are as follows: CSF Aβ42/Aβ40 = < 0.8 (CSF Aβ42/40 ratio x 10), CSF p-tau = > 60 pg/ml, and CSF t-tau = > 350 pg/ml.

**Table 6:** AD-driven MCI aAB biomarkers are capable of identifying preclinical AD pathology in otherwise cognitively normal (CDR = 0) STRIDE patients.

	NDC	STRIDE CDR = 0
**NDC**	24	1
**STRIDE CDR = 0**	1	17

**Table 6.** 18 STRIDE CDR = 0 were compared to 25 non-demented controls. Using 44 ADMCI aAB biomarkers, 17/18 CDR = 0 patients were identified as MCI, suggesting the presence of underlying AD pathology at the time of surgery; 24/25 non-demented controls were correctly classified as controls.

### ADMCI aAB biomarkers correlate strongly with CSF biomarkers indicative of the presence of AD pathology

We next sought to determine the degree of correlation between levels of the 44 ADMCI aAB biomarkers in the blood and levels of the more established CSF biomarkers. When CSF biomarker profiles of HFR patients with CDR = 0.5 were evaluated according to the A/T/N and corresponding NIA-AA classification system as described previously [47], 95.2% (20/21) of these patients were found to have abnormal CSF biomarker levels suggestive of underlying AD pathology, with one patient categorized as Suspected Non-Alzheimer’s Pathology (SNAP) (**Table 5**). Using the panel of 44 ADMCI aAB biomarkers, 95.2% (20/21) of patients with CDR = 0.5 were also identified as positive for MCI, with 19 of 20 (95.0%) patients overlapping with those identified as having abnormal CSF biomarker levels as determined by the A/T/N classification system. Together, this data suggests a high level of agreement among CDR scores at or above 0.5, abnormal levels of CSF biomarkers indicative of MCI, and the presence of ADMCI aAB biomarkers in identifying individuals with MCI due to early AD pathology in the STRIDE study.

## Discussion

In previously published work by our group, we showed that common, disease-associated changes in levels of specific autoantibodies (aABs) in blood, can be used as accurate blood-based biomarkers for detection and staging of neurodegenerative diseases such as AD, MCI due to AD, early and late stage Parkinson’s disease (PD), and multiple sclerosis [36–40]. In this study of older individuals from the STRIDE cohort who underwent hip fracture repair requiring surgery, we previously found a high prevalence of ongoing AD pathology as evidenced by abnormal levels of CSF Aβ42/40, total tau (t-tau) and phosphorylated tau (p-tau) in both cognitively normal (CDR = 0) and abnormal (CDR = 0.5 and >1.0) patients [42]. Using our panel of previously identified ADMCI aAB biomarkers, we sought to test the capability of these biomarkers to detect early AD-related neuropathology in the STRIDE cohort. Derived from studies carried out on ADNI subjects, this aAB panel has been used to distinguish cognitively normal individuals from patients with prodromal AD (MCI) due to the presence of ongoing, early stage AD-related neuropathology, as confirmed by measured levels of CSF biomarkers consistent with ongoing AD pathology [37].

The goals of the present study were to determine if a panel of previously identified ADMCI autoantibody biomarkers [37] could be utilized to distinguish STRIDE HFR participants with CDR = 0.5 from matched control subjects, and to test for any discernable relationship between CSF biomarkers of existing AD pathology and ADMCI biomarkers in the blood of the same subjects. We analyzed plasma samples from 39 HFR patients from the STRIDE cohort, as well as sera from 25 age-and sex-matched non-demented, non-surgical controls. The study data resulted in three main findings. First, we show that, in elderly HFR patients, the panel of 44 selected blood-based ADMCI aAB biomarkers can be used pre-surgically to detect the presence of early AD-related pathology in individuals presenting with cognitive impairment (as determine using CDR score) at the time of hospital admission with a high degree of accuracy. Second, these ADMCI aAB biomarkers are capable of identifying individual HFR patients who were cognitively normal (CDR = 0) at the time of surgery, but who later demonstrated accelerated post-surgical cognitive impairment and converted to either MCI (CDR = 0.5, n = 5) or AD (CDR≥1, n = 1) at one year following surgery. Lastly, the panel of ADMCI aAB biomarkers demonstrated exceptional agreement with the A/T/N classification system for CSF biomarkers (Aβ42/40, t-tau and p-tau) by successfully identifying 13/14 cognitively normal HFR patients (CDR = 0) as MCI, that had A/T/N designations consistent with preclinical AD. This finding suggests that the presence of ADMCI aAB biomarkers in the blood is directly linked to ongoing AD-related pathology and thus may be useful for preclinical detection of AD. Taken together, these results also suggest that a large proportion of individuals with hip fracture may have underlying AD pathology, and that ADMCI aAB biomarkers are potentially more sensitive than the modified CDR assessment and substantially less invasive than CSF biomarkers in identifying individual patients that may need specialized care in semi-urgent settings.

This study has several strengths. The first is that the panel of ADMCI aAB biomarkers used were derived from analysis of serum in a population of well-characterized individuals from the Alzheimer’s Disease Neuroimaging Initiative (ADNI). These individuals were diagnosed with MCI, shown to have a CDR = 0.5, and confirmed to have low CSF Aβ42, which is highly suggestive of ongoing early AD pathology. Another strength is the availability of pre-surgical CSF AD biomarker data from the patients who underwent hip fracture repair, which enabled biomarker confirmation of the underlying disease process along with clinical characterization. Additionally, the availability of longitudinal follow-up data allowed the examination of the utility of ADMCI aAB biomarkers as a tool to predict cognitive trajectory after surgery. Using established CSF biomarker values, 20/21 of the STRIDE HFR CDR = 0.5 patients were classified as preclinical AD using the A/T/N system. This was subsequently confirmed using the panel of 44 ADMCI aAB biomarkers, which also identified 19 of the same patients as MCI. Finally, a significant strength of this study is the validation of the panel of ADMCI aAB biomarkers in a completely independent patient cohort. This specific panel of biomarkers was previously tested in a cohort of 25 ADNI MCI subjects vs. 25 matched controls with high overall accuracy, sensitivity, and specificity [37]. Here, we demonstrate comparable accuracy, sensitivity, and specificity in the STRIDE subjects using 44 of the original 50 biomarkers from the panel.

This study also has several limitations, the foremost being the small sample size and clinical heterogeneity of the STRIDE HFR subject group. Intended as a “proof of concept” study, we acknowledge that although encouraging, our initial results warrant further studies in much larger and more diverse patient groups. Another limitation is that the determination of the CDR in the STRIDE subjects was largely informant based and not the formal process specified in the literature [42]. Due to the semi-urgent nature of traumatic hip fracture in an elderly population, a formal CDR determination is not feasible in this clinical setting. Another significant limitation introduced by the CSF biomarker assessment was that cutoff values for abnormal biomarkers were determined by population-based data, as well as previously published cutoff values. We acknowledge that the prevalence of abnormal biomarkers may vary depending on different laboratory assays and patient populations. Additional limitations include the divergent demographics of the Bioserve control population, which were 10 years younger on average, and also contained greater percentages of both female and Caucasian subjects when compared to the STRIDE cohort. Although an entirely age-matched control population would have been ideal, recent studies have demonstrated that significant proportions of age-matched individuals enrolled as controls in study groups such as ADNI displayed high baseline amounts of underlying AD pathology, based on comparative imaging data [50]. Due to the fact that a high percentage of subjects in the STRIDE cohort have demonstrated cognitive impairment consistent with early-stage AD or beyond, our use of a slightly younger control population helps to reduce the chance that these individuals will have underlying pre-symptomatic AD pathology, or if they do, less advanced pathology than their older STRIDE counterparts, as the incidence of AD rises substantially with each increasing decade of life [51]. In fact, we have found in our previous studies that “spiking” our elderly control groups with a small subset of samples from younger individuals increases classification accuracy using aAB biomarkers [36]. Lastly, the Bioserve control group also lacked CDR determinations as well as CSF biomarker assessments for comparison.

In conclusion, we report here a non-invasive, sensitive, and specific method to detect prodromal AD pre-surgically in individuals admitted into the hospital for hip fracture repair surgery. Early detection of AD-related pathology in this and other vulnerable patient populations has the potential to bring about improvements in peri- and postoperative patient care and management. In addition to early-stage detection, this panel of ADMCI aABs has also demonstrated the capability to identify individuals that were cognitively normal before surgery, but subsequently transitioned to MCI/AD within a year. Although preliminary, these results suggest the utility of blood-based aABs in preclinical detection of AD, perhaps years before the onset of clinical symptoms, thus paving the way for future therapies that could delay or halt disease progression altogether.

## Supporting information

S1 Table A panel of 44 selected AD-driven MCI aAB biomarkers derived from analysis of serum from a previous study using subjects enrolled in the Alzheimer’s Disease Neuroimaging Initiative (ADNI), for the diagnosis of MCI due to underlying AD etiology. (DOCX)
