# Supplementary material for: Detection of early-stage Alzheimer’s pathology using blood-based autoantibody biomarkers in elderly hip fracture repair patients
Source: PLoS One. 2019 Nov 15;14(11):e0225178. doi: 10.1371/journal.pone.0225178 (PMC6857922; doi:10.1371/journal.pone.0225178)
Supplement: S1 Table — (DOCX) [file pone.0225178.s001.docx]

**S1 Table 1:** **44 ADMCI aAB panel biomarkers.**

| Database ID | Protein Name |
| --- | --- |
| BC022098.1 | cDNA clone MGC:31944 IMAGE:4878869, complete cds |
| BC020233.1 | cDNA clone MGC:31936 IMAGE:4765518, complete cds |
| BC015833.1 | cDNA clone MGC:27152 IMAGE:4691630, complete cds |
| NM_032855.1 | hematopoietic SH2 domain containing (HSH2D) |
| BC030984.1 | cDNA clone MGC:32654 IMAGE:4701898, complete cds |
| NM_016207.2 | cleavage and polyadenylation specific factor 3, 73kDa (CPSF3) |
| BC016380.1 | cDNA clone MGC:27376 IMAGE:4688477, complete cds |
| BC051762.1 | Uncharacterized protein C20orf96 |
| XM_373800.2 | PREDICTED: Homo sapiens hypothetical LOC388528 (LOC388528) |
| BC053664.1 | zinc finger, FYVE domain containing 28 (ZFYVE28) |
| BC029796.1 | hypothetical protein BC014011 (LOC116349) |
| NM_000159.2 | glutaryl-Coenzyme A dehydrogenase (GCDH), nuclear gene encoding mitochondrial protein, transcript variant 1 |
| NM_001098.2 | aconitase 2, mitochondrial (ACO2), nuclear gene encoding mitochondrial protein |
| NM_014763.2 | mitochondrial ribosomal protein L19 (MRPL19), nuclear gene encoding mitochondrial protein |
| NM_018282.1 | Paraspeckle component 1 |
| XM_086879.4 | PREDICTED: Homo sapiens hypothetical LOC150371 (LOC150371) |
| BC104469.1 | Outer dense fiber protein 3-like protein 2 |
| BC032852.2 | melanoma antigen family B, 4 (MAGEB4) |
| NM_007255.1 | xylosylprotein beta 1,4-galactosyltransferase, polypeptide 7 (galactosyltransferase I) (B4GALT7) |
| NM_015891.2 | cell division cycle 40 homolog (S. cerevisiae) (CDC40) |
| NM_080548.1 | Tyrosine-protein phosphatase non-receptor type 6 |
| NM_006374.2 | serine/threonine kinase 25 (STE20 homolog, yeast) (STK25) |
| BC000468.1 | ubiquitin-conjugating enzyme E2 variant 1 (UBE2V1) |
| NM_182612.1 | Parkinson disease 7 domain containing 1 (PDDC1) |
| NM_001381.2 | docking protein 1, 62kDa (downstream of tyrosine kinase 1) (DOK1) |
| thyroglobulin | thyroglobulin |
| BC001304.1 | piccolo (presynaptic cytomatrix protein) (PCLO) |
| NM_003384.1 | vaccinia related kinase 1 (VRK1) |
| BC031068.1 | aminoadipate aminotransferase (AADAT) |
| BC030711.2 | Aprataxin and PNK-like factor |
| NM_024692.3 | CAP-GLY domain containing linker protein family, member 4 (CLIP4) |
| BC012423.1 | superoxide dismutase 2, mitochondrial (SOD2) |
| BC007852.1 | Serine/threonine-protein kinase 25 |
| NM_033377.1 | chorionic gonadotropin, beta polypeptide 1 [Source:RefSeq peptide;Acc:NP_203695] |
| NM_017451.1 | BAI1-associated protein 2 (BAIAP2), transcript variant 2 |
| BC056918.1 | glutathione S-transferase omega 2 (GSTO2) |
| NM_018357.2 | La ribonucleoprotein domain family, member 6 (LARP6), transcript variant 1 |
| NM_004732.1 | potassium voltage-gated channel, shaker-related subfamily, beta member 3 (KCNAB3) |
| BC030813.1 | cDNA clone MGC:22645 IMAGE:4700961, complete cds |
| NM_018039.2 | jumonji domain containing 2D (JMJD2D) |
| NM_022839.2 | mitochondrial ribosomal protein S11 (MRPS11), nuclear gene encoding mitochondrial protein, transcript variant 1 |
| NM_172160.1 | potassium voltage-gated channel, shaker-related subfamily, beta member 1 (KCNAB1), transcript variant 1 |
| BC017202.2 | Isovaleryl-CoA dehydrogenase, mitochondrial |
| BC017959.1 | chromosome 2 open reading frame 47 (C2orf47) |
